# Evaluation of NKp46 expression and cytokine production of decidual NK cells in women with recurrent pregnancy loss

**DOI:** 10.1002/rmb2.12478

**Published:** 2022-07-12

**Authors:** Mayu Yamamoto, Atsushi Fukui, Chuxian Mai, Shinichiro Saeki, Ryu Takayama, Yu Wakimoto, Ayano Yamaya, Joanne Kwak‐Kim, Hiroaki Shibahara

**Affiliations:** ^1^ Department of Obstetrics and Gynecology, School of Medicine Hyogo Medical University Nishinomiya Japan; ^2^ Clinical Sciences Department, Reproductive Medicine and Immunology, Obstetrics and Gynecology, Chicago Medical School Rosalind Franklin University of Medicine and Science Vernon Hills IL USA

**Keywords:** flow cytometry, natural killer cell, NKp46, receiver operating curve, recurrent pregnancy loss

## Abstract

**Purpose:**

NKp46, a receptor on NK cells, is involved in cytotoxicity and cytokine production. The authors aimed to evaluate the effect of NKp46 on decidual NK (dNK) cells during pregnancy and whether it can be a marker for immunological abnormalities in women with recurrent pregnancy loss (RPL).

**Methods:**

Flow‐cytometric analysis was made to assess NKp46 expression and intracellular cytokine production of dNK cells. The proportion of NKp46^+^ dNK cells was analyzed among RPL patients who aborted karyotypically normal pregnancies and those who either aborted karyotypically abnormal pregnancies or without genetic studies, and controls who were going through the induced abortion.

**Results:**

The **%**NKp46^+^ and %NKp46^bright^ dNK cells were significantly lower in the RPL women who aborted karyotypically normal pregnancies than in the control group. The %NKp46^bright^ dNK cells were significantly correlated with the NK1/NK2 ratio of dNK cells. The %NKp46^+^ dNK cell cutoff for RPL with immunological abnormalities was determined by the ROC curve analysis. In women with the low %NKp46^+^ dNK, NK1/NK2 ratios were significantly higher than those with the high.

**Conclusion:**

RPL patients with an immunological abnormality have decreased NKp46 expression and NK1 shift in dNK cells. NKp46 expression could be a marker for RPL of immunological abnormalities.

## Graphical abstract

Women with recurrent pregnancy losses with karyotypically normal pregnancy, which suggests pregnancy loss with immune etiologies, have decreased NKp46 expression (Decidual NKp46^bright^ less than 70.9% and NKp46^+^ less than 86.5%) and NK1 shift in dNK cells. NKp46 expression could be a marker for RPL of immune etiologies.

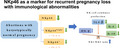

## 1 INTRODUCTION

Recurrent pregnancy loss (RPL) is defined as two or more clinical pregnancy losses.^1^ Approximately 60% of patients with RPL have repeated, unexplained miscarriages without common diagnoses, such as uterine malformation, the presence of antiphospholipid antibodies, thyroid dysfunction, or abnormal karyotypes in couples.^2^ Such unexplained miscarriages are possibly due to fetal chromosomal abnormalities or dysfunctional maternal immune responses.

An embryo is a semi‐allograft with paternal alloantigens recognized by the maternal immune system.^3^, ^4^ In early pregnancy, the inflammatory immune response is necessary for successful implantation. In particular, cytotoxic immune effectors at the maternal–fetal interface protect a fetus from pathogens while participating in tissue remodeling, angiogenesis, and preventing an excessive trophoblast invasion. Contrarily, dysregulated cytotoxic immune effectors may disturb pregnancy and induce pregnancy failures.^3^, ^5^, ^6^ Apart from cytotoxic immune effectors, regulatory T (Treg) cells also increase in early pregnancy. Treg cells are known to inhibit proliferation and cytokine production of both CD4^+^ and CD8^+^ T cells, immunoglobulin production by B cells, the cytotoxic activity of natural killer (NK) cells, and maturation of dendritic cells (DCs), resulting in the induction of tolerance.^7^, ^8^

The most abundant cells in the uterine endometrium are NK cells. The number of NK cells dramatically increases in the secretory phase and during early pregnancy^9^ to establish and maintain pregnancy. According to fluorescence staining intensity, NK cells express the CD56 receptor and can be subdivided into CD56^dim^ and CD56^bright^ cells. Approximately 90% of uterine NK (uNK) cells are CD56^bright^ cells, mainly involved in cytokine production,^10^ whereas approximately 90% of peripheral blood NK (pNK) cells are CD56^dim^ cells, involved in cytotoxicity.^11^ It has been reported that CD56^dim^ uNK cells increase in patients with RPL.^12^

NK cells express different kinds of receptors on their surface. NKp46, a natural cytotoxicity receptor (NCR), is involved in NK cell activation and functions in both cytotoxicity and cytokine production.^13^ NKp46 is a 46‐kDa type 1 membrane glycoprotein belonging to the immunoglobulin superfamily. It has two C2‐type Ig‐like domains in the extracellular portion and is associated with CD3ζ and FcεRIγ.^14^ When it recognizes and binds to its ligand, immunoreceptor tyrosine‐based activation motifs (ITAMs) are phosphorylated. They recruit Zap‐70 or SYK, leading to a cascade of reactions that ends with the intracellular release of calcium, inducing cytotoxicity and cytokine release.^13^ Hemagglutinin, a surface protein on influenza A and parainfluenza viruses, is an extrinsic ligand of NKp46.^15^ Intrinsic ligands of NKp46 are present in murine myeloma cell lines, human nevi, and melanoma cells.

Not only that, vimentin may be an intrinsic ligand.^16^ Vimentin, a 57‐kDa intermediate filament protein, is used as a cell differentiation marker. It is also expressed in the uterine endometrium and utilized to diagnose and grade cervical or endometrial cancer. NK cells kill activated CD4^+^ T cells through the NKp46/vimentin pathway.^17^ However, the role of the NKp46/vimentin pathway in reproduction has not been elucidated yet. In the case of uterine infection, NKG2A‐mediated negative signals, which control NKp46‐mediated cytolytic function, might be abrogated by viral immune evasion mechanisms, leading to the absence or diminished expression of its HLA‐E‐specific ligand,^18^ and the upregulation of the NKp46 specific ligand. Such NKp46‐mediated cytotoxic activity and the NKp30‐mediated secretion of inflammatory cytokines by decidual NK (dNK) may decrease the number of infected uterine cells.^19^ Recently, we reported that the numbers of NKp46^+^ CD16^−^ NK cells were low in patients with higher CD56^dim^/CD16^+^ NK cells, accompanied by an NK2 shift.^20^ We have also reported that NKp46^dim^ NK cells may be involved in NK cell cytotoxicities, whereas NKp46^bright^ NK cells may be involved in cytokine production, suggesting that NKp46 could be a predictive marker for immune tolerance in pregnancy.^21^

There are two types of NK cells: NK1 cells producing inflammatory (Type 1) cytokines, such as IFN‐γ and TNF‐α, and NK2 cells producing anti‐inflammatory (Type 2) cytokines, such as IL‐4 and IL‐10.^22^ NK cells show polarities in their cytokine secretion profiles, comparable to the polarities of T helper (Th) cells.^23^ There is an increase in type 2 cytokine production in the uterus in a healthy pregnancy, called the NK2 shift^24^ or Th2 shift.^25^ Decreased type1 inflammatory response protects the fetus, while NK1 shift has been reported in women with RPL and recurrent implantation failure (RIF) after in vitro fertilization and embryo transfer cycles.^26^ We have previously reported that NKp46 expression was low on the surface of pNK and/or uNK cells in women with various forms of reproductive failures, such as RPL and RIF.^27^, ^28^, ^29^ Besides, we have also reported that low expression of activating receptors on NKp46^+^ uNK cells is more prevalent in high‐risk women.^20^ Thus, NKp46 plays an important role in reproduction through cytotoxicity and cytokine production. However, the detailed mechanism remains unknown and NKp46 expression on dNK cells in patients with RPL has not been investigated. Therefore, we aim to explore the role of NKp46^+^ dNK cells in patients with RPL and whether NKp46 can be used to detect RPL with immunological abnormalities by analyzing the relationship between the expression of NKp46 and cytokine production of dNK cells.

## 2 MATERIALS AND METHODS

### 2.1 Ethical approval and study participants

We enrolled patients at the Department of Obstetrics and Gynecology, Hyogo Medical University Hospital, between April 2018 and December 2020. All participants provided written, informed consent before enrolling in the study, approved by the Hyogo Medical University Institutional Review Board, and complied with the Declaration of Helsinki (2013).

dNK cells were collected from 43 women undergoing dilatation and curettage (Table 1), including women with two or more pregnancy losses (the RPL group, *n* = 31) and those who had an induced abortion due to medical conditions or abnormal fetal karyotype (the control group, *n* = 12). Patients who had an artificial abortion for maternal protection or who had an abortion for the first time owing to abnormal fetal karyotype were put in the control group. The RPL group was subdivided into two subgroups; those who had abortions with karyotypically normal pregnancy (*n* = 11) and those with karyotypically abnormal pregnancy or without genetic studies (*n* = 20; including 11 patients who did not perform chorionic karyotyping).

**TABLE 1:** Age, number of pregnancies, deliveries, spontaneous abortions, and induced abortions of participants

	RPL group with karyotypically normal pregnancies	RPL group without karyotypically normal pregnancies	Controls	*p*†
	(*n* = 11)	(*n* = 20)	(*n* = 12)	
Age (years)	34.2 ± 4.0	36.5 ± 5.7	33.6 ± 6.9	N.S.
Number of pregnancies	2.6 ± 0.8	3.9 ± 2.2	2.1 ± 0.9	<0.05
		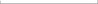		
Number of deliveries	0.2 ± 0.4	1.0 ± 0.8	0.8 ± 0.9	<0.05
	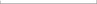			
Number of spontaneous abortions	2.3 ± 0.8	2.8 ± 1.4	0.7 ± 0.7	<0.05
		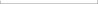		
	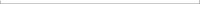			
Number of induced abortions	0.1 ± 0.3	0.2 ± 0.5	0.5 ± 0.5	<0.05
	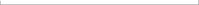			
Pregnancy days before abortion (days)	65.6 ± 9.8	63.4 ± 6.2	66.1 ± 10.2	N.S.

†One‐way analysis of variance (ANOVA) was implemented. Data are presented as mean ± SD.

*Two groups with significant differences by Tukey's test (*p* < 0.05).

### 2.2 Preparation of decidual leukocytes

Decidual cells were placed in sterile Roswell Park Memorial Institute (RPMI) 1640 medium (Thermo Fisher Scientific, Waltham, MA, USA) supplemented with 10% fetal calf serum, 1% penicillin, and 1% streptomycin (Thermo Fisher Scientific). After the macroscopic exclusion of blood, samples were minced by micro scissors, then mechanically disrupted by a gentleMACS™ Dissociator (Miltenyi Biotec, Bergisch Gladbach, Germany) to create decidual single‐cell suspensions. The final concentration was adjusted to 5 × 10^6^ cells/mL.

### 2.3 Measurement of surface antigens on dNK cells

Surface antigens on dNK cells were stained using the following monoclonal antibodies (Table S1): anti‐CD45‐APC‐H7 (Clone 2D1) and anti‐CD56‐Alexa Fluor 488 (Clone B159) (both from BD Bioscience, San Jose, CA, USA); and anti‐CD3‐APC (Clone UCHT1), anti‐CD16‐BV510 (Clone 3G8), and anti‐NKp46‐BV421 (Clone 9E2) (all from BioLegend Inc., San Diego, CA, USA). Negative control and appropriate isotype control for each antibody were performed simultaneously with the surface antigen staining. Monoclonal antibodies were incubated with 100 μl of decidual cell suspension for 20 min at 4°C in the dark, then lysed and fixed, washed twice in phosphate‐buffered saline (PBS), and finally, resuspended in 0.25 ml PBS for subsequent flowcytometric analysis.

### 2.4 Flowcytometric analysis

**FIGURE 1:**
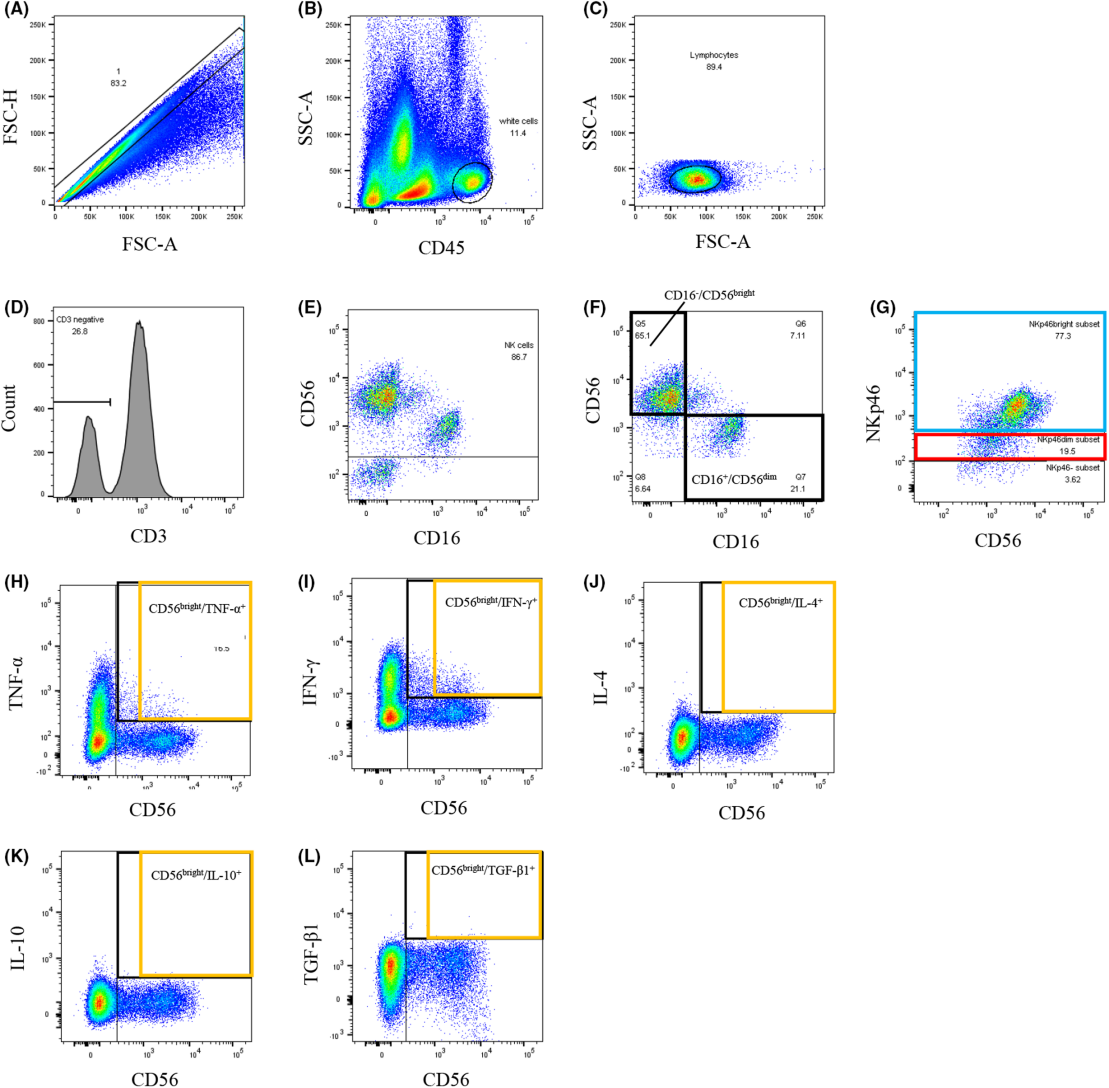
Gating strategies of decidual NK cells and representative dot‐plots of NKp46‐expressing decidual NK cells and cytokine‐producing decidual NK cells. (A) Gating strategy for removal of doublets by forward scatter (FSC) height and area parameters. (B) Anti‐CD45 APC‐H7 was used to detect uterine endometrial leukocytes and exclude other cells. (C) Characteristic forward and side scatter parameters were used to detect uterine endometrial lymphocytes and exclude other cells. (D) Anti‐CD3 APC was used to remove NKT cells (CD3^+^/CD56^+^). (E) Decidual NK cells were determined as CD56^+^ cells. (F) CD56^+^ cells are classified as CD56^dim^ and CD56^bright^ cells, and the percentages of CD16^+^/CD56^dim^ and CD16^−^/CD56^bright^ are calculated. (G) NKp46^+^ cells are classified as NKp46^dim^ and NKp46^bright^ cells. Representative dot plots of cytokine production by decidual NK cells: (H) TNF‐α‐producing CD56^bright^ NK cells; (I) IFN‐γ‐producing CD56^bright^ NK cells; (J) IL‐4‐producing CD56^bright^ NK cells; (K) IL‐10‐producing CD56^bright^ NK cells; and (L) TGF‐β1‐producing CD56^bright^ NK cells

Immunofluorescence staining, 5‐color flowcytometric analysis of surface antigens staining, and 7‐color cytokine staining of dNK cells were performed using LSRFortessaX‐20 (BD Bioscience). BD FACS Diva software (BD Bioscience) was used for full‐list‐mode data storage and recovery. FlowJo (Flow Jo LLC, Ashland, OR, USA) was used for analysis. Doublets were excluded using forward scatter height and area parameters. The gate was set on anti‐CD45‐APC‐H7 positive events followed by lymphocyte regions using characteristic forward, and side scatters parameters. CD3^−^/CD56^+^ cells were gated as dNK cells (Figure 1A–D). At least 3 × 10^4^ lymphocytes were collected in each sample. We distinguished CD56^dim^ and CD56^bright^ NK cells and NKp46^dim^ and NKp46^bright^ cells based on fluorescence staining intensity of CD56 and NKp46, respectively (Figure 1E–G).

### 2.5 Intracellular cytokine production by dNK cells

Decidual cell suspensions (200 μl) were stimulated with 25 ng/mL phorbol 12‐myristate 13‐acetate (PMA), 1 μM ionomycin, and 10 μg/mL brefeldin A (all from Sigma–Aldrich Inc., St. Louis, MO, USA) for 4 h at 37°C in a 5% CO_2_ humidified incubator; then washed with PBS and stained for 20 min at 4 °C in the dark with anti‐CD45‐APC‐H7 and anti‐CD56‐Alexa Fluor 488 antibodies. Subsequently, cells were washed and fixed with 250 μl Cytofix/Cytoperm (BD Bioscience) and then stained for 30 min with anti‐TNF‐α‐BV421 (Clone Mab11), anti‐IFN‐γ‐PE‐Cy7 (Clone 4S.B3), anti‐IL‐4‐PerCP‐Cy5 (Clone MP4‐25D2), anti‐IL‐10‐APC (Clone JES3‐19F1), and anti‐TGF‐β‐PE (Clone TW4‐2F8) (all from BioLegend, Inc.), with negative control and appropriate isotype control for each antibody (Table S1B). Finally, the decidual cell suspensions were washed twice with 1× Perm Wash Solution (BD Bioscience) and resuspended in 0.5 ml PBS for flow cytometry (Figure 1H–L). Intracellular cytokine production of dNK cells was analyzed in 26 of 43 patients; the remaining 17 patients did not have a sufficient amount of decidual tissue.

### 2.6 Statistical analysis

Data were analyzed using SPSS 23 (IBM Corp., Armonk, NY, USA). One‐way analysis of variance (ANOVA) was used to compare the distribution of age, number of pregnancies, deliveries, miscarriages, and pregnancy days among the three groups; then, Tukey's test was performed between the two groups. Data are presented as the mean ± standard deviation (SD). Differences in surface antigenic expression, intracellular cytokine production, and the NK1/NK2 cytokine ratios among the three groups were analyzed using the Kruskal–Wallis test. Differences between the two groups were analyzed using the Dunn test; the data are presented as the median with interquartile ranges. Differences were considered significant at *p* < 0.05. Correlations between the percentage of NKp46^bright^ dNK cells and cytokine production ratios of CD56^bright^ dNK cells were analyzed by Spearman's correlation coefficient test and considered significant when r > 0.4 and *p* < 0.05. Receiver operating characteristic (ROC) curve analysis was performed using SPSS; the area under the ROC curves was calculated for RPL with karyotypically normal pregnancies, and the cutoff value for %NKp46^+^ dNK was determined.

## 3 RESULTS

### 3.1 Patient characteristics

Table 1 shows the obstetrical histories of the study patients and controls. The number of pregnancies in the RPL group without karyotypically normal pregnancies was significantly higher than in the control group (*p* < 0.05). The number of previous deliveries was significantly lower in the RPL with karyotypically normal pregnancies than in the RPL without karyotypically normal pregnancies (*p* < 0.05). The number of spontaneous abortions in the RPL group with and without karyotypically normal pregnancies was significantly higher than in the control group (*p* < 0.05, respectively). The number of induced abortions in the RPL group with karyotypically normal pregnancies was significantly lower than in the control group (*p* < 0.05). There was no significant difference in gestational days at abortion among the three groups.

### 3.2 Expression of surface antigens on dNK cells

#### 3.2.1 CD16 and CD56 co‐expression on dNK cells

Representative dot‐plots of CD16 and CD56 co‐expressing dNK cells are shown in Figure 1E,F. The %CD16^+^/CD56^dim^ and %CD16^−^/CD56^bright^ cells were not significantly different among the three groups (Table 2).

**TABLE 2:** Expression of CD16 and CD56 on dNK cells

	RPL group with karyotypically normal pregnancies (*n* = 11)		RPL group without karyotypically normal pregnancies (*n* = 20)		Controls (*n* = 12)		*p*†"/>
	Median	Interquartile range	Median	Interquartile range	Median	Interquartile range	
CD16^+^/CD56^dim^	19.84	[10.89–21.28]	12.76	[5.99–22.81]	16.33	[11.41–26.35]	N.S.
CD16^−^/CD56^bright^	53.90	[49.00–68.00]	60.20	[39.32–79.60]	61.05	[45.18–72.85]	N.S.

^†^Kruskal–Wallis test was implemented. Data are presented as median [Interquartile ranges] and N.S., not significant.

#### 3.2.2 NKp46 expression on dNK cells

**FIGURE 2:**
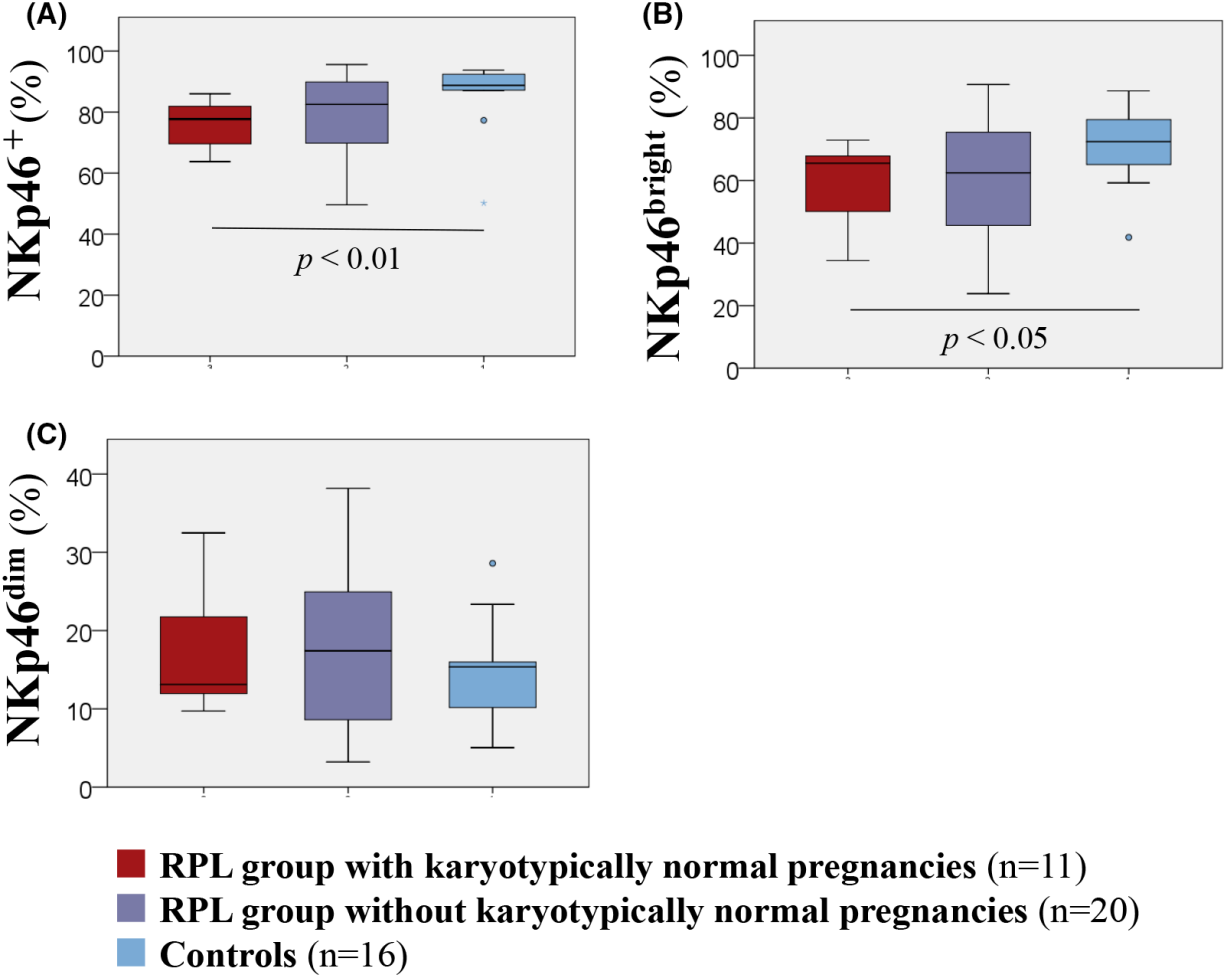
NKp46^+^, NKp46^dim^, and NKp46^bright^ expression on decidual NK cells. Differences in (A) NKp46^+^ dNK cells, (B) NKp46^bright^ dNK cells, and (C) NKp46^dim^ dNK cells among the RPL group with karyotypically normal pregnancies, the RPL group without karyotypically normal pregnancies, and the controls. Box and whisker plots: bar (horizontal line) = median; box = 25th and 75th percentiles; whiskers = extend to the extreme values. Red bars show the RPL group with karyotypically normal pregnancies; purple bars show the RPL group without karyotypically normal pregnancies; blue bars show the controls. Differences between the three groups were analyzed by the Kruskal–Wallis test; the Dunn test was performed between each of the two groups

Representative dot‐plots of NKp46 expressed on dNK cells of each group are shown in Figure S1A–C. The %NKp46^+^ dNK cells in the RPL group with karyotypically normal pregnancies (77.72%, interquartile range [IQR]; 69.59–81.92) was significantly lower than that in the controls (88.77%, IQR; 82.27–91.98) (*p* < 0.01; Figure 2A). There was no significant difference in the percentage of NKp46^+^ dNK cells between the RPL group without karyotypically normal pregnancies and the control groups. The percentage of NKp46^bright^ dNK cells in the RPL group with karyotypically normal pregnancies (65.51% [50.11–67.85]) was significantly lower than that in the control group (72.43%, IQR; 66.66–78.42)(*p* < 0.05; Figure 2B), with no significant difference between the RPL group without karyotypically normal pregnancies and the control group. The differences in the %NKp46^dim^ dNK cells among the three groups (Figure 2C) were insignificant.

### 3.3 Cytokine production of dNK cells and its correlation with NKp46 expression

#### 3.3.1 Cytokine production and NK1/NK2 ratios of dNK cells

Representative dot‐plots of cytokine production of dNK cells are shown in Figure 1H–L. There were no significant differences in the percentages of TNF‐α, IFN‐γ, IL‐4, IL‐10, or TGF‐β1 producing dNK cells (Table 3A) or in the TNF‐α/IL‐4, IFN‐γ/IL‐4, TNF‐α/IL‐10, and IFN‐γ/IL‐10 producing CD56^bright^ dNK cell ratios among the three groups (Table 3B).

**TABLE 3:** Intracellular cytokine production of dNK cells

	RPL group with karyotypically normal pregnancies (*n* = 7)			RPL group without karyotypically normal pregnancies (*n* = 10)			Controls (*n* = 9)		^†^
	Median	Interquartile range	*p* ^‡^	Median	Interquartile range	*p* ^‡^	Median	Interquartile range	
(A) The percentages of TNF‐α‐, IFN‐γ‐, IL‐4‐, IL‐10‐, and TGF‐β1‐producing CD56^bright^ dNK cells in three study groups									
TNF‐α^+^	8.12	[4.05–8.49]	0.80	7.00	[3.71–8.93]	0.76	5.25	[4.81–6.06]	
IFN‐γ^+^	7.42	[5.94–9.82]	1.00	6.65	[3.50–12.20]	0.35	5.66	[5.03–8.15]	
IL‐4^+^	1.54	[1.37–1.99]	0.49	2.09	[1.00–3.52]	0.25	2.48	[1.40–2.79]	
IL‐10^+^	1.85	[1.62–2.09]	0.73	1.34	[0.90–2.08]	0.84	1.96	[1.11–2.61]	
TGF‐β1^+^	2.35	[2.22–2.87]	0.61	3.42	[2.57–3.94]	0.41	2.85	[2.40–3.74]	
(B) TNF‐α/IL‐4, IFN‐γ/IL‐4, TNF‐α/IL‐10, and IFN‐γ/IL‐10 ratios of CD56^bright^ dNK cells in three study groups									
TNFα/IL‐4	3.36	[2.48–6.31]	0.49	3.21	[1.85–5.42]	0.35	2.71	[1.41–3.75]	
IFN‐γ/IL‐4	5.66	[2.79–6.58]	0.61	3.84	[1.82–7.35]	0.21	2.30	[2.00–5.82]	
TNF‐α/IL‐10	4.39	[1.93–5.86]	0.67	4.68	[1.99–6.96]	0.61	3.42	[2.41–4.21]	
IFN‐γ/IL‐10	5.88	[2.62–6.02]	0.73	4.97	[2.22–11.42]	0.41	3.51	[2.19–4.88]	

^†^No significant difference was shown in Kruskal‐Wallis test among the three groups (*p* ≧ 0.05).

^‡^No significant difference was shown with controls in the Dunn test (*p* ≧ 0.05).

Data are presented as median [Interquartile range].

#### 3.3.2 Correlations between NKp46 ^+^ dNK cells and cytokine production and NK1/NK2 ratios of dNK cells

There were significant negative correlations between the NKp46^+^ dNK cells, NKp46^bright^ dNK cells, and IL‐4 (*r* = 0.57, *p* < 0.05; *r* = 0.72, *p* < 0.05; respectively), and IL‐10 producing CD56^bright^ dNK cell (*r* = 0.47, *p* < 0.05; *r* = 0.65, *p* < 0.05; respectively). In addition, there were significant positive correlations between the %NKp46^+^ dNK cells and TGF‐β1 producing CD56^bright^ dNK cells (*r* = 0.42, *p* < 0.05). However, there was no significant correlation between the percentages of NKp46^+^ dNK cells, NKp46^bright^ dNK cells, and TNF‐α or IFN‐γ producing CD56^bright^ dNK cells (Table S2A). The %NKp46^bright^ dNK cells showed significant negative correlations with TNF‐α/IL‐10 ratio (*r* = −0.42, *p* < 0.05), and IFN‐γ/IL‐10 ratio (*r* = −0.41, *p* < 0.05) of CD56^bright^ dNK cells, but not with TNF‐α/IL‐4 or IFN‐γ/IL‐4 ratios of CD56^bright^ dNK cells (Figure S2, Table S2B). There was no significant correlation between the %NKp46^+^ dNK cells and the NK1/NK2 ratios of CD56^bright^ dNK cells (Table S2B).

### 3.4 NKp46 expression thresholds and cytokine production of dNK cells

#### 3.4.1 NKp46^bright^ threshold and cytokine production of dNK cells

**FIGURE 3:**
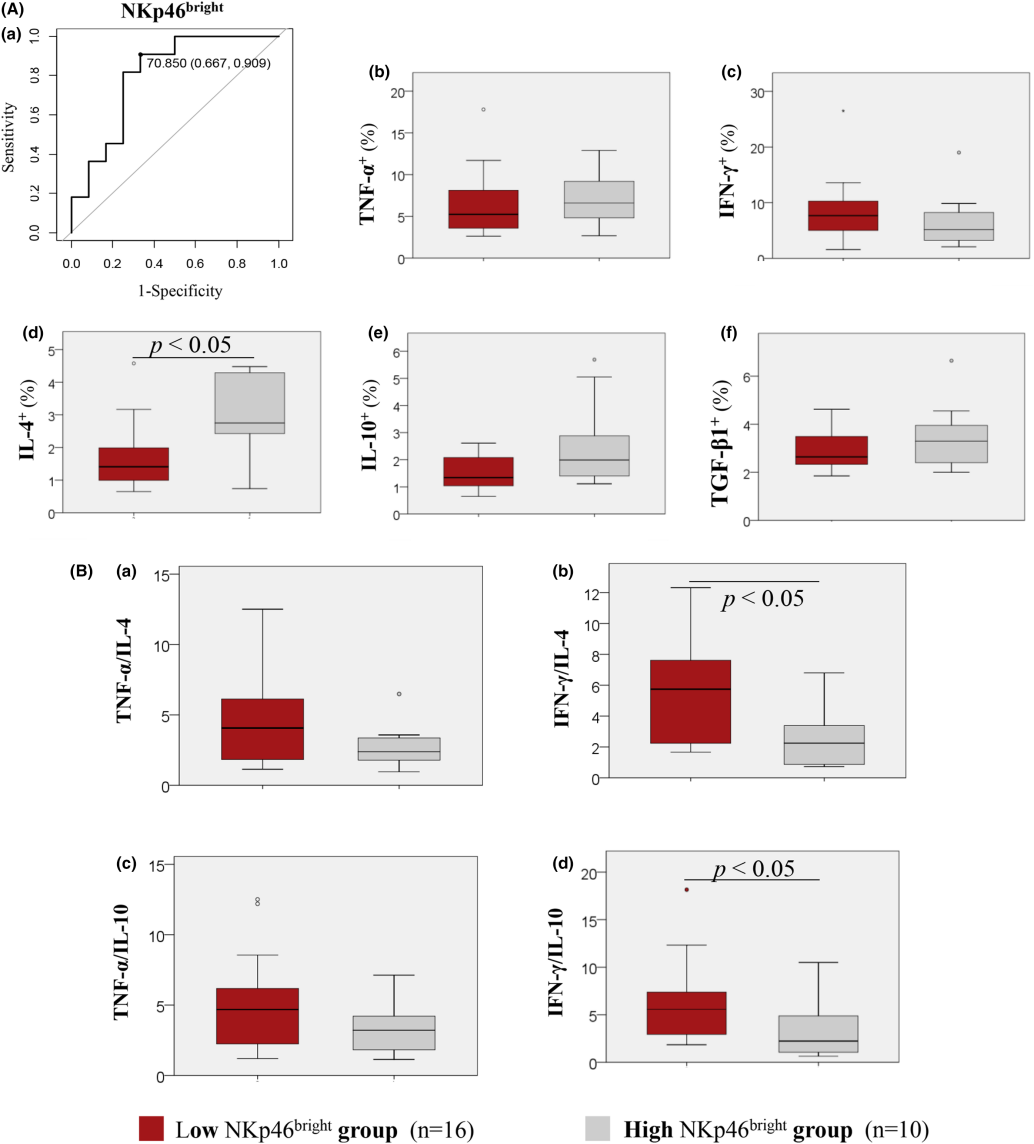
Characteristics of NKp46^bright^ dNK cells. (A) Receiver operating characteristic (ROC) curve of NKp46^bright^ and cytokine production of CD56^bright^ dNK cells. (B) NK1/NK2 ratios of CD56^bright^ dNK cells were assessed by the NKp46^bright^ threshold. (A) Cytokine production from CD56^bright^ dNK cells compared using an NKp46^bright^ threshold of 70.85%. (a) ROC curves for determining (d) the NKp46^+^ dNK cell proportion threshold based on recurrent pregnancy loss (RPL) with normal chorionic villi. Vertical axis = sensitivity; horizontal axis = 1 − specificity: Diagonal = reference line: Points on the curves = thresholds (%). ROC curve analysis was performed based on nonparametric assumptions with a true area = 0.5 for the null hypothesis. Differences in the percentage of CD56^bright^ dNK cells producing (b) TNF‐α, (c) IFN‐γ, (d) IL‐4, (e) IL‐10, and (f) TGF‐β1 between the low NKp46^bright^ and high NKp46^bright^ groups. (B) Cytokine production ratios of CD56^bright^ dNK cells compared using an NKp46^bright^ threshold of 70.85%. Differences in (a) TNF‐α/IL‐4, (b) IFN‐γ/IL‐4, (c) TNF‐α/IL‐10, and (d) IFN‐γ/IL‐10 ratios of CD56^bright^ dNK cells between the low NKp46^bright^ and high NKp46^bright^ groups. Box and whisker plots: bar (horizontal line) = median; box = 25th and 75th percentiles; whiskers = extend to the extreme values. Red bars show the low NKp46^bright^ group. Gray bars show the high NKp46^bright^ group. Differences between the two groups were analyzed using the Mann–Whitney *U*‐test; differences were considered significant at *p* < 0.05

According to ROC curve analyses, based on the RPL with karyotypically normal pregnancies, the RPL threshold of the %NKp46^bright^ dNK fraction was 70.85%, showing 66.7% sensitivity and 90.9% specificity (Figure 3Aa), with 80% area under the curve (AUC; Table 4A), 88.9% positive predictive value (PPV), and 71.4% negative predictive value (NPV) (Table 4B). Based on the %NKp46^bright^ dNK threshold (70.85%), patients were divided into a low NKp46^bright^ dNK group (NKp46^bright^ dNK cells <70.85%; *n* = 16) and high NKp46^bright^ dNK group (NKp46^bright^ dNK cells ≥70.85%; *n* = 10). The percentage of IL‐4 producing CD56^bright^ dNK cells in the low NKp46^bright^ dNK group (2.75% [2.44–4.13]) was significantly lower than that in the high NKp46^bright^ dNK group (1.41% [1.01–1.92]; *p* < 0.05; Figure 3Ad). The percentages of TNF‐α, IFN‐γ, IL‐10, or TGF‐β1 producing CD56^bright^ dNK cells were comparable between the low NKp46^bright^ and high NKp46^bright^ groups (Figure 3Ab–c,e–f). IFN‐γ/IL‐4 and IFN‐γ/IL‐10 ratios in CD56^bright^ dNK cells were significantly higher in the low NKp46^bright^ dNK group (5.74 [2.26–7.30] and 5.58 [3.17–7.18], respectively) than that in the high NKp46^bright^ dNK group (2.25 [0.95–3.12] and 2.23 [1.26–4.76], respectively; *p* < 0.05; Figure 3Bb,d). The TNF‐α/IL‐4 and TNF‐α/IL‐10 ratios of CD56^bright^ dNK cells were comparable between the low and high NKp46^bright^ groups (Figure 3Ba,c).

**TABLE 4:** NKp46 expression threshold to detect recurrent pregnancy loss (RPL) with immunological abnormalities by receiver operating characteristic (ROC) curve analysis. (A) Area under the curve (AUC), standard error (SE), asymptotic P value, and asymptotic confidence interval for NKp46 expression thresholds. (B) Sensitivity, specificity, positive predictive value (PPV), and negative predictive value (NPV) for the NKp46 expression threshold

(A)						
	AUC	S.E.	Asymptotic *p* value	Asymptotic confidence interval		
NKp46^+^ threshold	0.87*	0.09	<0.05	0.70	–	1.00
NKp46^bright^ threshold	0.80*	0.10	<0.05	0.62	–	0.99

(B)					
	Cut‐off point (%)	Sensitivity (%)	Specificity (%)	PPV (%)	NPV (%)
NKp46^+^ threshold	86.52	83.3	100	100	84.62
NKp46^bright^ threshold	70.85	66.7	90.9	88.89	71.43

*Null hypothesis (true AUC = 0.5) rejected in ROC curve analysis (*p* < 0.05).

#### 3.4.2 NKp46 ^+^ threshold and cytokine production of dNK cells

**FIGURE 4:**
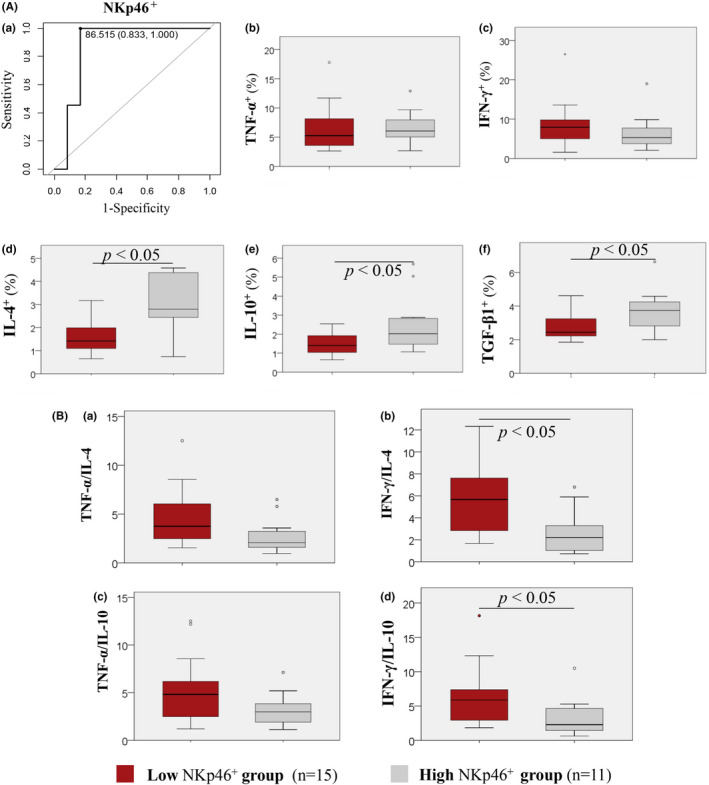
Characteristics of NKp46^+^ dNK cells. (A) Receiver operating characteristic (ROC) curve of NKp46^+^ and cytokine production of CD56^bright^ dNK cells. (B) NK1/NK2 ratios of CD56^bright^ dNK cells were assessed by the NKp46^+^ threshold. (A) Cytokine production from CD56^bright^ dNK cells using an NKp46^+^ threshold of 86.52%. (a) The ROC curves for determining the (d) NKp46^+^ dNK cell proportion threshold based on recurrent pregnancy loss (RPL) with normal chorionic villi. Vertical axis = sensitivity; horizontal axis = 1 − specificity: Diagonal = reference line: Points on the curves = thresholds (%). ROC curve analysis was performed based on nonparametric assumptions with a true area = 0.5 for the null hypothesis. Differences in the percentage of CD56^bright^ dNK cells producing (b) TNF‐α, (c) IFN‐γ, (d) IL‐4, (e) IL‐10, and (f) TGF‐β1 between the low NKp46^+^ and high NKp46^+^ groups. (B) Cytokine production ratio of CD56^bright^ dNK cells using an NKp46^+^ threshold of 86.52%. Differences in (a) TNF‐α/IL‐4, (b) IFN‐γ/IL‐4, (c) TNF‐α/IL‐10, and (d) IFN‐γ/IL‐10 ratios of CD56^bright^ dNK cells between the low NKp46^+^ and high NKp46^+^ groups. Box and whisker plots: bar (horizontal line) = median; box = 25th and 75th percentiles; whiskers = extend to the extreme values. Red bars show the low NKp46^+^ group. Gray bars show the high NKp46^+^ group. Differences between the two groups were analyzed using the Mann–Whitney *U*‐test; differences were considered significant at *p* < 0.05

According to ROC curve analyses, based on the RPL with karyotypically normal pregnancies, the RPL threshold of the %NKp46^+^ dNK was 86.52%, showing 83.3% sensitivity and 100% specificity (Figure 4Aa), with 87.0% AUC (Table 4A), 100% PPV, and 84.62% NPV (Table 4B). Based on the NKp46^+^ dNK threshold of 86.52%, patients were divided into the low NKp46^+^ group (NKp46^+^ dNK cells <86.52%; *n* = 15) and high NKp46^+^ group (NKp46^+^ dNK cells ≥86.52% *n* = 11). In the low NKp46^+^ group, the percentages of IL‐4‐producing CD56^bright^ dNK (1.42% [1.10–1.99]), IL‐10‐producing dNK (1.40% [1.04–1.92]), and TGF‐β‐producing dNK (2.44% [2.22–3.25]) cells were significantly lower than that in the high NKp46^+^ group (2.79% [2.44–4.38]; 2.02% [1.48–2.83]; 3.74% [2.82–4.25]); respectively (*p* < 0.05; Figure 4Ad–f). The percentages of TNF‐α‐, or IFN‐γ‐producing CD56^bright^ dNK cells were comparable between the low and high NKp46^+^ groups (Figure 4Aa–b). The IFN‐γ/IL‐4 and IFN‐γ/IL‐10 ratios were significantly higher in the low NKp46^+^ group (5.66 [2.84–7.61]; 5.88 [2.94–7.30]) than in the high NKp46^+^ group (2.06 [1.60–3.23]; 2.28 [1.45–4.64]; respectively; *p* < 0.05; Figure 4Bb,d). The TNF‐α/IL‐4 and TNF‐α/IL‐10 ratios were comparable between the low and high NKp46^+^ groups (Figure 4Ba,c).

## 4 DISCUSSION

This study explored immunological abnormalities in RPL women with karyotypically normal pregnancies by analyzing NKp46 expression on dNK cells and cytokine production of dNK cells. NKp46 is a natural cytotoxicity receptor (NCR) expressed on NK cells. In addition, it is expressed on ILCs and γδ T cells.^30^ NKp46^dim^ and NKp46^bright^ uNK cells have different roles; NKp46^dim^ uNK cells primarily have a cytotoxic function, while NKp46^bright^ uNK cells are involved in cytokine production.^21^ However, its relationship to reproduction remains unclear.

In this study, we report that decreased %NKp46^+^ dNK cells were associated with RPL without karyotypically normal pregnancies than in the control group. This contradicts previous studies, reporting increased NKp46 expression on NK cells in reproductive failures, such as RPL for dNK cells^31^ or repeated implantation failures for peripheral blood NK cells.^32^ However, this study is consistent with our previous studies, demonstrating the decreased expression of NKp46, especially for the NKp46^bright^ fraction, on pNK and uNK cells in RPL,^27^, ^33^ suggesting a relationship between reproductive failures and NCRs, including NKp30, NKp44, and NKp46. Besides, in this study, %NKp46^bright^ dNK cells were decreased in the RPL group with karyotypically normal pregnancies compared with the control group. Therefore, the previously reported decrease in NKp46 uNK and pNK cell levels might have been due to the decreased proportion of NKp46^bright^ NK cells.

We used ROC curve analysis to establish an appropriate %NKp46 dNK threshold for RPL with immune etiologies. The %NKp46^+^ dNK cell threshold of 86.52% showed a higher PPV and NPV than those of the %NKp46^bright^ dNK cell threshold. Indeed, the brightness of CD56 is correlated with low NK cell cytotoxicity. In the present study, there was a decrease in the percentage of NKp46^bright^ and NKp46^+^ cells in the RPL group with karyotypically normal pregnancies. The NK1/NK2 ratios correlate with the percentages of NKp46^bright^ cells rather than NKp46^+^ cells, suggesting that %NKp46^bright^ has a function in cytokine production (Table S2 and Figure S2). NKp46^bright^ cells are related to cytokine production, and in the RPL group with karyotypically normal pregnancies, the reduction of NKp46^bright^ cells is thought to cause abnormal cytokine production (NK1 shift), leading to miscarriage. However, as shown in Table 4, the percentage of NKp46^+^ cells would be a more useful marker with higher PPV and NPV than the percentage of NKp46^bight^ cells when we seek a cutoff for whether or not RPL with karyotypically normal pregnancies occurs. In other words, NKp46^birght^ cell abnormalities have functional importance in aberrant cytokine production, leading to miscarriage, and NKp46^+^ cells abnormality has importance in diagnosing a subgroup of RPL with karyotypically normal pregnancies (RPL with immune abnormalities). Therefore, the %NKp46^+^ dNK cell threshold seems to be more appropriate for identifying RPL with immunological abnormalities. In addition, there are two more reasons. Firstly, NKp46^+^ dNK cells can be easily distinguished from NKp46^−^ dNK cells; however, it may be difficult to distinguish NKp46bright from NKp46dim dNK cells clearly. Secondly, the main function of NKp46^dim^ dNK cells remains unknown. When considering these facts, NKp46^+^ dNK cells seem to be a better biomarker than NKp46^bright^ dNK cells to predict RPL with immunological abnormalities.

During the cytokine production analysis of CD56^bright^ dNK cells (Table 3), higher TNF‐α and IFN‐γ production and lower IL‐4, IL‐10, and TGF‐1β production were observed in the RPL group with karyotypically normal pregnancies compared with the control group, although differences were not significant. NK1 cytokines such as TNF‐α and IFN‐γ contribute to a successful pregnancy with TGF‐β1 by participating in spiral artery remodeling and establishing vascular connections between the fetus and the mother; however, excessive inflammatory cytokine production can be harmful to the placenta. Therefore, a balance between NK1 and NK2 cytokine production and a timely shift to NK2 cytokine production is critical to a successful pregnancy.^23^ In this study, TNF‐α/IL‐4, TNF‐α/IL‐10, IFN‐γ/IL‐4, and IFN‐γ/IL‐10 producing dNK cell ratios appeared to shift to NK1 in the RPL group with karyotypically normal pregnancies.

NKp46^bright^ dNK cells had negative correlations with TNF‐α/IL‐10 and IFN‐γ/IL‐10 producing CD56^bright^ dNK cell ratios (Figure S2). In addition, we compared cytokine production based on the %NKp46^+^ and %NKp46^bright^ dNK cell thresholds (Figures 3 and 4). IL‐4 production was significantly lower in the low NKp46^bright^ dNK group than in the high NKp46^bright^ dNK group. IL‐4, IL‐10, and TGF‐β1 production were significantly lower in the low NKp46^+^ dNK than in the high NKp46^+^ dNK groups. Both the low NKp46^bright^ and NKp46^+^ dNK groups had higher IFN‐γ/IL‐4 and IFN‐γ/IL‐10 ratios than the high NKp46^bright^ and NKp46^+^ dNK groups. Thus, the decreased NKp46^bright^ and NKp46^+^ dNK cells may contribute to the NK1 shift. It has been reported that activated T cells express more vimentin than others. Vimentin binds to NKp46 on NK cells and inhibits their cytotoxicity.^17^ Reduced NKp46^+^ dNK cells in RPL patients may result in immune dysregulation with increased or sustained NK activity or cytotoxicity and NK1 shift. Further studies are needed.

This study has some limitations. Firstly, to measure cytokine production of NKp46^+^ dNK cells directly, we measured NKp46^+^ dNK cells and cytokines producing dNK cells separately because NKp46 expression would be diminished by the stimulation of PMA, ionomycin, and brefeldin‐A (data not shown). One way to analyze intracellular cytokine production of NKp46^+^ dNK cells is to isolate NKp46^+^ dNK cells by magnetic microbeads before stimulating cytokine production. Noteworthily, more cytokine production was reported in NKp46^bright^ cells than in NKp46^dim^ cells.^34^ Hence, further studies are needed to investigate cytokine production of NKp46^+^ dNK cell subsets. Secondly, not every aborted tissue was tested for karyotype in this study. Patients with untested fetal karyotypes (*n* = 11) were assigned to the RPL group without karyotypically normal pregnancies (*n* = 20), which may decrease the data reliability. Although it is important to investigate the genetic cause of miscarriages, karyotyping was not done in every case due to medical and socioeconomic reasons. Thirdly, it is unclear whether changes in NKp46 expression have a causal relationship with miscarriages or epiphenomenon. If it is the consequence of a miscarriage, the cytotoxicity of NKp46^dim^ dNK cells is increased with decreased NKp46^bright^ dNK cells and increased NKp46^dim^ dNK cells in the RPL group with karyotypically normal pregnancies. In this study, we demonstrated the decreased NKp46^+^ dNK cells in the RPL group with karyotypically normal pregnancies. Hence, it is speculated that NKp46 expression can be causally related to miscarriages. Lastly, since the NKp46 expression was studied in dNK cells, it cannot be monitored during pregnancies. Whether pNK cells have the same pattern of NKp46 expression has not been studied well, although some reports demonstrate differences in the expression of NKp46 in decidua and peripheral NK cells.^35^, ^36^, ^37^

In conclusion, in RPL patients with karyotypically normal pregnancies, NKp46 expression on dNK cells is decreased, and cytokine production of dNK cells is shifted to NK1. It is suggested that measuring decidual NKp46 expression may help predict RPL with immunological abnormalities. Determining the cause of RPL with unknown risk factors and investigating the cause of each miscarriage, such as immune disorders and abnormal fetal karyotype, are important factors for personalized treatments of RPL patients.

## CONFLICT OF INTEREST

The authors declare no conflict of interest.

## HUMAN RIGHTS STATEMENTS AND INFORMED CONSENT

This study was approved by the institutional review board of the Hyogo Medical University (IRB number 2871). Human rights statements and informed consent: all procedures followed were in accordance with the ethical standards of the responsible committee on human experimentation (institutional and national) and with the Helsinki Declaration of 1964 and its later amendments. Informed consent was obtained from all patients being included in the study.

## ANIMAL RIGHTS

This article does not contain any studies with animal subjects performed by any authors.

## Supporting information


Figure S1‐S2

Table S1‐S2

## References

[1] Practice committee of the American Society for reproductive medicine. Electronic address aao. Definitions of infertility and recurrent pregnancy loss: a committee opinion. Fertil Steril. 2020;113(3):533–5. 32115183 10.1016/j.fertnstert.2019.11.025

[2] Morita K , Ono Y , Takeshita T , Sugi T , Fujii T , Yamada H , et al. Risk factors and outcomes of recurrent pregnancy loss in Japan. J Obstet Gynaecol Res. 2019;45(10):1997–2006. 31397532 10.1111/jog.14083

[3] Papuchova H , Meissner TB , Li Q , Strominger JL , Tilburgs T . The dual role of HLA‐C in tolerance and immunity at the maternal‐fetal Interface. Front Immunol. 2019;10:2730. 31921098 10.3389/fimmu.2019.02730 PMC6913657

[4] Tilburgs T , Strominger JL . CD8+ effector T cells at the fetal‐maternal interface, balancing fetal tolerance and antiviral immunity. Am J Reprod Immunol. 2013;69(4):395–407. 23432707 10.1111/aji.12094 PMC3711858

[5] Jauniaux E , Burton GJ . Pathophysiology of placenta Accreta Spectrum disorders: a review of current findings. Clin Obstet Gynecol. 2018;61(4):743–54. 30299280 10.1097/GRF.0000000000000392

[6] Acar N , Ustunel I , Demir R . Uterine natural killer (uNK) cells and their missions during pregnancy: a review. Acta Histochem. 2011;113(2):82–91. 20047753 10.1016/j.acthis.2009.12.001

[7] Sakaguchi S . Naturally arising Foxp3‐expressing CD25 + CD4+ regulatory T cells in immunological tolerance to self and non‐self. Nat Immunol. 2005;6(4):345–52. 15785760 10.1038/ni1178

[8] Akbar AN , Vukmanovic‐Stejic M , Taams LS , Macallan DC . The dynamic co‐evolution of memory and regulatory CD4+ T cells in the periphery. Nat Rev Immunol. 2007;7(3):231–7. 17318234 10.1038/nri2037

[9] Lee JY , Lee M , Lee SK . Role of endometrial immune cells in implantation. Clin Exp Reprod Med. 2011;38(3):119–25. 22384430 10.5653/cerm.2011.38.3.119 PMC3283071

[10] Robertson MJ , Ritz J . Biology and clinical relevance of human natural killer cells. Blood. 1990;76(12):2421–38. 2265240

[11] Lanier LL , Le AM , Civin CI , Loken MR , Phillips JH . The relationship of CD16 (Leu‐11) and Leu‐19 (NKH‐1) antigen expression on human peripheral blood NK cells and cytotoxic T lymphocytes. J Immunol. 1986;136(12):4480–6. 3086432

[12] Lachapelle MH , Miron P , Hemmings R , Roy DC . Endometrial T, B, and NK cells in patients with recurrent spontaneous abortion. Altered profile and pregnancy outcome. J Immunol. 1996;156(10):4027–34. 8621945

[13] Orange JS , Ballas ZK . Natural killer cells in human health and disease. Clin Immunol. 2006;118(1):1–10. 16337194 10.1016/j.clim.2005.10.011

[14] Biassoni R , Cantoni C , Marras D , Giron‐Michel J , Falco M , Moretta L , et al. Human natural killer cell receptors: insights into their molecular function and structure. J Cell Mol Med. 2003;7(4):376–87. 14754506 10.1111/j.1582-4934.2003.tb00240.x PMC6740120

[15] Arnon TI , Lev M , Katz G , Chernobrov Y , Porgador A , Mandelboim O . Recognition of viral hemagglutinins by NKp44 but not by NKp30. Eur J Immunol. 2001;31(9):2680–9. 11536166 10.1002/1521-4141(200109)31:9<2680::aid-immu2680>3.0.co;2-a

[16] Garg A , Barnes PF , Porgador A , Roy S , Wu S , Nanda JS , et al. Vimentin expressed on mycobacterium tuberculosis‐infected human monocytes is involved in binding to the NKp46 receptor. J Immunol. 2006;177(9):6192–8. 17056548 10.4049/jimmunol.177.9.6192

[17] Chong WP , Zhou J , Law HK , Tu W , Lau YL . Natural killer cells become tolerogenic after interaction with apoptotic cells. Eur J Immunol. 2010;40(6):1718–27. 20391434 10.1002/eji.200939768

[18] Brooks CR , Elliott T , Parham P , Khakoo SI . The inhibitory receptor NKG2A determines lysis of vaccinia virus‐infected autologous targets by NK cells. J Immunol. 2006;176(2):1141–7. 16434388 10.4049/jimmunol.176.2.1141

[19] El Costa H , Casemayou A , Aguerre‐Girr M , Rabot M , Berrebi A , Parant O , et al. Critical and differential roles of NKp46‐ and NKp30‐activating receptors expressed by uterine NK cells in early pregnancy. J Immunol. 2008;181(5):3009–17. 18713971 10.4049/jimmunol.181.5.3009

[20] Takeyama R , Fukui A , Mai C , Yamamoto M , Saeki S , Yamaya A , et al. Co‐expression of NKp46 with activating or inhibitory receptors on, and cytokine production by, uterine endometrial NK cells in recurrent pregnancy loss. J Reprod Immunol. 2021;145:103324. 33930666 10.1016/j.jri.2021.103324

[21] Mai C , Fukui A , Takeyama R , Yamamoto M , Saeki S , Yamaya A , et al. NK cells that differ in expression of NKp46 might play different roles in endometrium. J Reprod Immunol. 2021;147:103367. 34464905 10.1016/j.jri.2021.103367

[22] Mosmann TR , Cherwinski H , Bond MW , Giedlin MA , Coffman RL . Two types of murine helper T cell clone. I. Definition according to profiles of lymphokine activities and secreted proteins. 1986. J Immunol. 2005;175(1):5–14. 15972624

[23] Peritt D , Robertson S , Gri G , Showe L , Aste‐Amezaga M , Trinchieri G . Differentiation of human NK cells into NK1 and NK2 subsets. J Immunol. 1998;161(11):5821–4. 9834059

[24] Borzychowski AM , Croy BA , Chan WL , Redman CW , Sargent IL . Changes in systemic type 1 and type 2 immunity in normal pregnancy and pre‐eclampsia may be mediated by natural killer cells. Eur J Immunol. 2005;35(10):3054–63. 16134082 10.1002/eji.200425929

[25] Saito S . Cytokine network at the feto‐maternal interface. Journal of Reproductive Immunology. 2000;47(2):87–103. 10924744 10.1016/s0165-0378(00)00060-7

[26] Fukui A , Kwak‐Kim J , Ntrivalas E , Gilman‐Sachs A , Lee SK , Beaman K . Intracellular cytokine expression of peripheral blood natural killer cell subsets in women with recurrent spontaneous abortions and implantation failures. Fertil Steril. 2008;89(1):157–65. 17482605 10.1016/j.fertnstert.2007.02.012

[27] Fukui A , Ntrivalas E , Gilman‐Sachs A , Kwak‐Kim J , Lee SK , Levine R , et al. Expression of natural cytotoxicity receptors and a2V‐ATPase on peripheral blood NK cell subsets in women with recurrent spontaneous abortions and implantation failures. Am J Reprod Immunol. 2006;56(5–6):312–20. 17076675 10.1111/j.1600-0897.2006.00431.x

[28] Fukui A , Ntrivalas E , Fukuhara R , Fujii S , Mizunuma H , Gilman‐Sachs A , et al. Correlation between natural cytotoxicity receptors and intracellular cytokine expression of peripheral blood NK cells in women with recurrent pregnancy losses and implantation failures. Am J Reprod Immunol. 2009;62(6):371–80. 19821805 10.1111/j.1600-0897.2009.00750.x

[29] Fukui A , Funamizu A , Fukuhara R , Shibahara H . Expression of natural cytotoxicity receptors and cytokine production on endometrial natural killer cells in women with recurrent pregnancy loss or implantation failure, and the expression of natural cytotoxicity receptors on peripheral blood natural killer cells in pregnant women with a history of recurrent pregnancy loss. J Obstet Gynaecol Res. 2017;43(11):1678–86. 28815854 10.1111/jog.13448

[30] Barrow AD , Martin CJ , Colonna M . The natural cytotoxicity receptors in health and disease. Front Immunol. 2019;10:909. 31134055 10.3389/fimmu.2019.00909 PMC6514059

[31] Zhang Y , Zhao A , Wang X , Shi G , Jin H , Lin Q . Expressions of natural cytotoxicity receptors and NKG2D on decidual natural killer cells in patients having spontaneous abortions. Fertil Steril. 2008;90(5):1931–7. 18023431 10.1016/j.fertnstert.2007.08.009

[32] Dons'koi BV , Osypchuk DV , Chernyshov VP , Khazhylenko KG . Expression of natural cytotoxicity receptor NKp46 on peripheral blood natural killer cells in women with a history of recurrent implantation failures. J Obstet Gynaecol Res. 2021;47(3):1009–15. 33368832 10.1111/jog.14631

[33] Fukui A , Funamizu A , Yokota M , Yamada K , Nakamua R , Fukuhara R , et al. Uterine and circulating natural killer cells and their roles in women with recurrent pregnancy loss, implantation failure and preeclampsia. J Reprod Immunol. 2011;90(1):105–10. 21632120 10.1016/j.jri.2011.04.006

[34] Yokota M , Fukui A , Funamizu A , Nakamura R , Kamoi M , Fuchinoue K , et al. Role of NKp46 expression in cytokine production by CD56‐positive NK cells in the peripheral blood and the uterine endometrium. Am J Reprod Immunol. 2013;69(3):202–11. 23311919 10.1111/aji.12062

[35] Moffett A , Hiby SE , Sharkey AM . The role of the maternal immune system in the regulation of human birthweight. Philos Trans R Soc Lond B Biol Sci. 2015;370(1663):20140071. 25602075 10.1098/rstb.2014.0071 PMC4305172

[36] Hanna J , Goldman‐Wohl D , Hamani Y , Avraham I , Greenfield C , Natanson‐Yaron S , et al. Decidual NK cells regulate key developmental processes at the human fetal‐maternal interface. Nat Med. 2006;12(9):1065–74. 16892062 10.1038/nm1452

[37] Koopman LA , Kopcow HD , Rybalov B , Boyson JE , Orange JS , Schatz F , et al. Human decidual natural killer cells are a unique NK cell subset with immunomodulatory potential. J Exp Med. 2003;198(8):1201–12. 14568979 10.1084/jem.20030305 PMC2194228

