# Supplementary material for: Evaluation of NKp46 expression and cytokine production of decidual NK cells in women with recurrent pregnancy loss
Source: Reprod Med Biol. 2022 Jul 12;21(1):e12478. doi: 10.1002/rmb2.12478 (PMC9275167; doi:10.1002/rmb2.12478)
Supplement: Supplementary file 1 — Figure S1‐S2 [file RMB2-21-e12478-s002.pptx]

## Slide 1
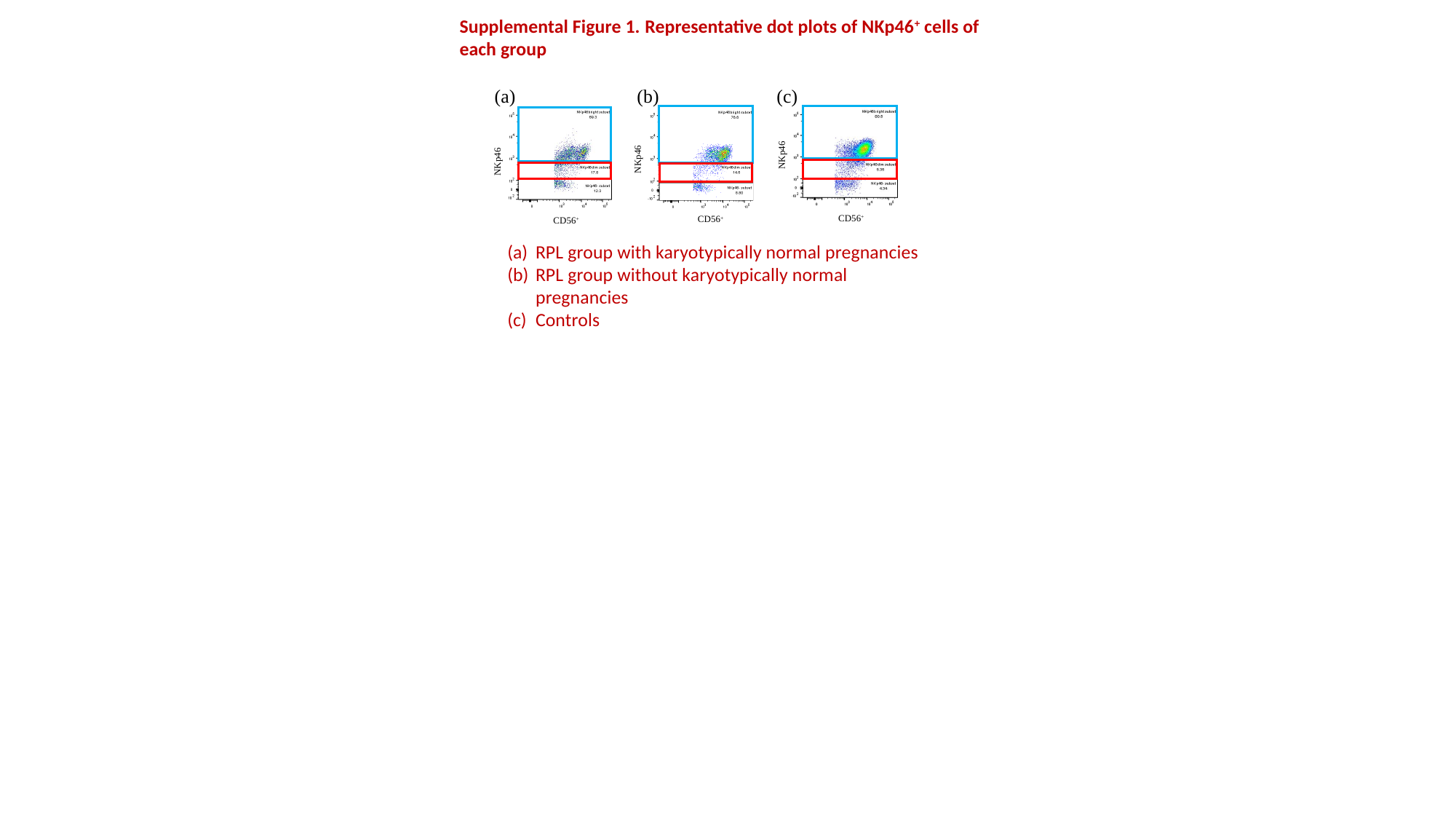

Supplemental Figure 1. Representative dot plots of NKp46+ cells of each group
(a)
(b)
(c)
NKp46
NKp46
NKp46
CD56+
CD56+
CD56+
RPL group with karyotypically normal pregnancies
RPL group without karyotypically normal pregnancies
Controls

## Slide 2
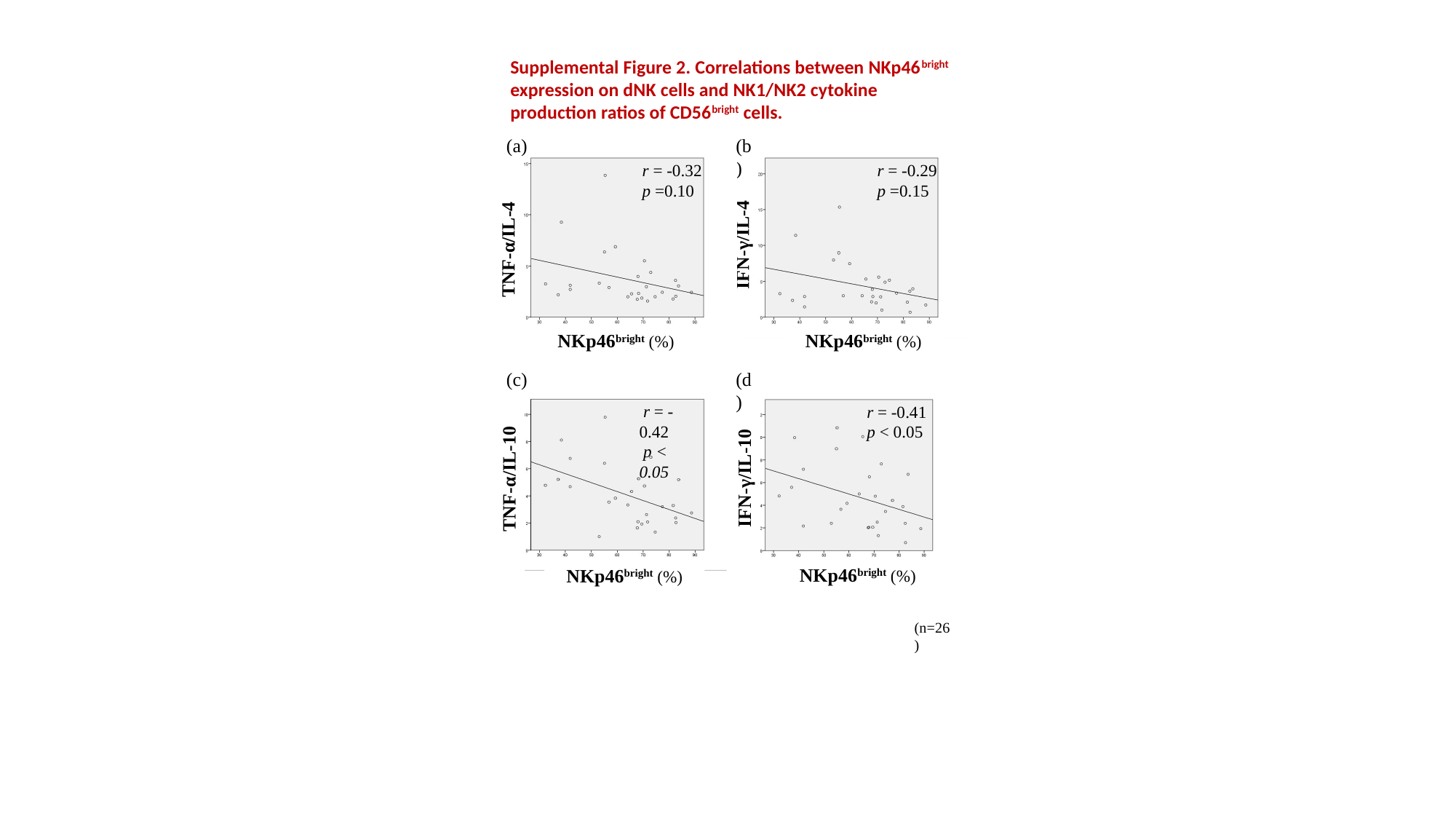

Supplemental Figure 2. Correlations between NKp46bright expression on dNK cells and NK1/NK2 cytokine production ratios of CD56bright cells.
(a)
(b)
 r = -0.29
 p =0.15
 r = -0.32
 p =0.10
IFN-γ/IL-4
TNF-α/IL-4
NKp46bright (%)
NKp46bright (%)
(c)
(d)
 r = -0.42
 p < 0.05
 r = -0.41
 p < 0.05
IFN-γ/IL-10
TNF-α/IL-10
NKp46bright (%)
NKp46bright (%)
 (n=26)
